# Utility of Downstream Biomarkers to Assess and Optimize Intranasal Delivery of Oxytocin

**DOI:** 10.3390/pharmaceutics14061178

**Published:** 2022-05-31

**Authors:** Megan DuBois, Angela Tseng, Sunday M. Francis, Ann F. Haynos, Carol B. Peterson, Suma Jacob

**Affiliations:** Department of Psychiatry and Behavioral Sciences, University of Minnesota, Minneapolis, MN 55455, USA; duboi175@umn.edu (M.D.); atseng@umn.edu (A.T.); sfrancis@umn.edu (S.M.F.); afhaynos@umn.edu (A.F.H.); peter161@umn.edu (C.B.P.)

**Keywords:** oxytocin, intranasal, urine, plasma, autism, anorexia, cluster analysis, drug delivery

## Abstract

Oxytocin (OT), a mammalian neurohormone associated with social cognition and behavior, can be administered in its synthetic form intranasally (IN) and impact brain chemistry and behavior. IN-OT shows potential as a noninvasive intervention for disorders characterized by social challenges, e.g., autism spectrum disorder (ASD) and anorexia nervosa (AN). To evaluate IN-OT’s efficacy, we must quantify OT uptake, availability, and clearance; thus, we assessed OT levels in urine (uOT) before and after participants (26 ASD, 7 AN, and 7 healthy controls) received 40 IU IN-OT or placebo across two sessions using double-blind, placebo-controlled crossover designs. We also measured uOT and plasma (pOT) levels in a subset of participants to compare the two sampling methods. We found significantly higher uOT and pOT following intranasal delivery of active compound versus placebo, but analyses yielded larger effect sizes and more clearly differentiated pre–post-OT levels for uOT than pOT. Further, we applied a two-step cluster (TSC), blinded backward-chaining approach to determine whether active/placebo groups could be identified by uOT and pOT change alone; uOT levels may serve as an accessible and accurate systemic biomarker for OT dose–response. Future studies will explore whether uOT levels correlate directly with behavioral targets to improve dosing for therapeutic goals.

## 1. Introduction

In recent decades, interest in the therapeutic potential of intranasal drugs has been on the rise; noninvasive delivery of neurohormones that target the central nervous system (CNS) has gained attention because this approach may effectively alter brain chemistry and behavior [1,2]. Oxytocin (OT), a key neuropeptide associated with social behavior and cognition [3,4,5], is of particular interest given the myriad neurodevelopmental and psychiatric conditions in which these domains are impacted. Autism spectrum disorder (ASD), a neurodevelopmental disorder (NDD) characterized by deficits in social communication, is thus a logical subject of investigation. Relatedly, the use of exogenous OT has also been suggested as a potential treatment for anorexia nervosa (AN), an eating disorder often comorbid with ASD or obsessive compulsive disorder, as well as other relevant disorders, such as social anxiety [6,7]. Similar to ASD, AN is marked by rigidity, high anxiety, and social difficulties [8,9]. Intranasal oxytocin (IN-OT) has been shown to dampen salivary cortisol response to stressors, especially in clinical populations, and may improve treatment outcomes by acting on the hypothalamic–pituitary–adrenal (HPA) axis [8,9]. However, ASD clinical trials testing both chronic and single-dose IN-OT have yielded inconsistent results due, in part, to the use of varying outcome measures, e.g., caregiver-reported social responsiveness [10,11,12], facial emotion recognition task performance [13], or scales used for pharmacological trials [14,15].

At present, nearly 200 IN-OT trials are registered on ClinicalTrials.gov [16]. In addition to ASD and AN, IN-OT has also been studied in schizophrenia for its potential effects on social cognition and symptom severity [17,18]. These trials suggest that oxytocin administration in schizophrenic adults affects certain facets of social cognition compared with placebo (e.g., brain activity during fMRI tasks), but, similar to autism trials, the overall effect on symptoms and cognition is unclear. Other targets for investigational IN-OT include post-traumatic stress disorder, (PTSD), Borderline Personality Disorder (BPD), social anxiety disorder, obesity, and postpartum depression [19,20,21,22,23]. Yet, variability in design and techniques across studies and populations hinder our ability to synthesize the extant literature. Herein we examine the methodological considerations (e.g., delivery and timing) that can impact results.

### 1.1. Intranasal Delivery Compared with Other Noninvasive Methods (e.g., Oral, Transdermal)

Although multiple avenues of delivery have been used to administer pharmacological treatments, multiple toxicological, anatomical, and physiological factors are critical to consider when developing drug protocols. Particularly in trials that involve vulnerable populations (e.g., pediatric or cognitively-impaired) or protocols requiring doses [24,25], noninvasive methods (e.g., oral, transdermal, and intranasal) are more tenable than more invasive and aversive approaches (e.g., requiring needle sticks). Yet, despite the overall convenience of drug administration via the oral route, several drawbacks must be factored into procedural designs. A number of variables can impact oral drug absorption, such as drug solubility, mucosal permeability, and stability in the gastrointestinal tract environment; pharmacokinetics can be affected by presystemic or first-pass metabolism and bioavailability of the drug, as well as potential drug–drug interactions [25].

Alternatively, whereas both oral and transdermal administration approaches show slow onset of action, transdermal delivery circumvents first-pass effects and its benefits can be long-acting. However, while transdermal patches have increased in usage, one key disadvantage is that only certain drugs (e.g., small molecules) are candidates for patch delivery [26]. Intranasal drug administration offers another course of action that is fast-acting relative to transdermal administration and can be applied effectively to treatment of CNS disorders because molecules are absorbed through the trigeminal and olfactory pathways, have higher bioavailability, and enable some drugs to pass through the blood–brain barrier [27]. Intranasal drugs are frequently administered for use in allergy and cold symptoms; intranasal glucocorticoids are among the most common and effective treatments for chronic rhinosinusitis [28]. Moreover, although both intranasal and transdermal drug deliveries are well-tolerated by patients, some data suggest that intranasal administration may be preferable to a transdermal patch or gel. For example, in a randomized, within-subjects comparison of intranasal and patch estradiol in postmenopausal women, 66% of women chose to continue with intranasal estradiol instead of transdermal after the conclusion of the study [29]. While both intranasal and transdermal forms of the drug were effective at reducing symptoms and resulted in comparable occurrences of adverse events, participants endorsed higher levels of satisfaction with intranasal delivery, reporting it to be more efficacious, discreet, and quicker. Similarly, in an intranasal testosterone therapy (TTh) study of hypogonadal men who were either TTh-naive or had experience with topical TTh, intranasal TTh and topical TTh were found to be equally effective, while the majority of participants found intranasal TTh to be more convenient [30]. These findings support the use of intranasal drug administration as an accessible, fast-acting, and safe method for treatment and everyday symptom reduction.

### 1.2. Biomarkers to Determine Effects of Intranasal Delivery

Despite ongoing debate on the mechanisms involved in how intranasal drug delivery targets behavioral outcomes and how best to measure uptake and action (i.e., centrally versus peripherally), research continues without consensus on best practices. While some compounds delivered intranasally can traverse the blood–brain barrier and affect CNS function directly [27,31], the lumbar punctures required to measure drug levels in cerebrospinal fluid (CSF) are highly invasive. Similarly, assessing drug levels in blood plasma requires invasive blood draws, procedures that may be aversive to children as well as adults. Consequently, when feasible, noninvasive methods have become prevalent both clinically and in research.

Saliva assays are one such method used to determine peripheral levels, but it should be noted that reliability and correlation between plasma and saliva are inconsistent [32,33,34]. Moreover, sample collection protocols are critical to consider when assessing measurement accuracy. For example, portions of the drug may remain in the nasopharynx after administration and this dosing discrepancy must be incorporated into calculations. Drug administration and saliva collection procedures must account for dosage levels and temporal dynamics of salivary OT concentrations; lack of consistency in duration between baseline and post-administration measures can impact assay results significantly [35]. Further, saliva measures may be precluded in disorders that affect salivary glands or saliva production; for example, in eating disorders, purging has been shown to impact the validity of saliva samples [36].

**Figure 1:**
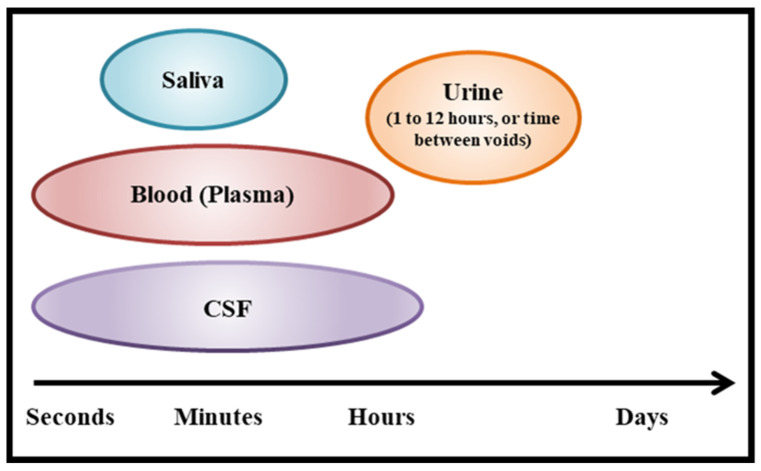
Timescales captured by common bio-sample types.

Consequently, we investigated whether an alternative approach, measuring peripheral OT levels in urine (uOT), might yield more consistent and reliable results. One key advantage of assaying for uOT is the relative ease of sample collection as long as levels are calibrated for fluid intake/excretion variability. Since urine is pooled in the bladder over a period of hours, levels reflect total volumes over time instead of transient peaks with rapid clearance. Although uOT may not replace time course measurements in CSF or plasma (Figure 1), this technique affords us a noninvasive methodology that can be used to ascertain the impact of IN-OT dosage levels on targeted behaviors. For example, researchers have reported pre–post associations between uOT and social outcomes, including children’s responding to vocal, nonverbal, and physical comfort, men’s visual search for infant and adult faces, and the relationship anxiety or parenting stress among mothers [37,38,39].

Whereas a handful of publications have included measures of both uOT and pOT, results have been inconsistent. For example, while social and behavioral outcomes have been associated with both uOT and pOT levels in some studies of typical development and ASD [37,39,40,41], research with AN participants has demonstrated poor correspondence between OT levels collected via plasma and urine (e.g., Hoffman et al., 2012) [36]. Further, studies that compare pOT with uOT levels directly to assess quality of measurement are lacking. Thus, in the present study, our primary objective was to evaluate and contrast uOT and pOT sampling in ASD participants as a means to detect IN-OT outcomes. We predicted that the noninvasive nature of uOT sampling would not only be better tolerated generally by our participants than the use of blood draws for pOT, but we also hypothesized that uOT measures would yield more precise metrics of IN-OT uptake than plasma measures. Additionally, we extended our investigation of uOT to both ASD and AN participants in order to gauge applicability within a larger clinical sample characterized by OT-related phenotypes.

## 2. Materials and Methods

### 2.1. Participants

For this study, we collected data from two separate clinical populations (ASD and AN) and a healthy control (HC) group matched to AN participants according to age, sex, and race/ethnicity. ASD participants (mean age: 14.42 ± 5.06 years; N = 26; 3F) were recruited through the University of Illinois at Chicago (UIC) and the University of Minnesota (UMN). Clinicians trained in DSM-IV-TR [42] classification of autism confirmed diagnoses for our ASD sample. Individuals on a medication schedule were not excluded if they had been on a stable routine for a minimum of 3 months before starting the study and if they were able maintain stability for the duration of their participation. Individuals did not meet inclusion criteria if they had any medical concerns (e.g., significant nasal pathology, drug/alcohol abuse, cardiovascular, endocrine, gastrointestinal, hematological, hepatic, and respiratory disease) that might preclude completion of study components [43].

Female AN and HC participants (mean age 29.64 ± 11.10 years; N = 14; 7AN) were also recruited from UMN; all AN participants met DSM-5 criteria for AN as confirmed by the SCID-5-RV [44]. Exclusion criteria included pregnancy or lactation, acute suicidality, history of neurological disorder or injury, and current substance use disorder, psychosis, mania, other medical instability, or nasal pathology. AN participants were allowed to take psychoactive medications (e.g., antidepressants and antipsychotics) but they had to have been on a stable dose for 6 weeks prior to the first study visit. Exclusion criteria for HC participants were presence of current DSM-5 diagnosis, current or past eating disorder diagnosis, and use of psychoactive medications.

Mean age across all included participants was 19.75 ± 10.56 years (N = 40; F: 17). All protocols were approved by their respective Institutional Review Boards and conducted in accordance with the Code of Federal Regulations (Title 45, Part 46), National Institutes of Health, and the Office for Protection from Research Risks of the US Federal Government [45].

### 2.2. Design

In a double-blind, placebo-controlled, crossover challenge study of single-dose IN-OT versus placebo, ASD participants were asked to complete two study sessions scheduled approximately two weeks apart. Placebo or IN-OT (weight-adjusted standardization based on 40 IU/67 kg dose or 0.6 IU/kg/dose goal) were administered to participants at each visit and the order of active and placebo administration sessions was counterbalanced across participants and visits (1,2). Participants provided both urine and blood plasma samples before and after drug administration at each session (see details in protocol) and they were scheduled for their follow-up visit at the same time of day as the first session to account for everyday fluctuations in endogenous OT. In addition, in order to mitigate confounds that might be introduced by circadian variability and changes in diet, participants were asked to maintain similar sleep–wake–meal schedules, and consume similar foods and beverages for both study sessions. Participants were allowed to drink water to alleviate thirst and/or if they required hydration to provide a urine sample. AN and HC participants followed a nearly identical, highly time-standardized protocol, with a double-blind, placebo-controlled, crossover challenge design. However, AN and HC participant sessions were separated by approximately one week rather than two and only provided urine samples.

### 2.3. Drug

The IN-OT formulation, Syntocinon, was manufactured by Novartis (Basel, Switzerland) and the placebo formulation was sourced from Novartis but manufactured by Advantage Pharmaceuticals Inc. (Rocklin, CA, USA). Previous studies of IN-OT single-dose tolerability [43,46] and chronic dosing safety in ASD [43] informed our dosage level selection. Both UIC and UMN investigational drug pharmacies were responsible for storing and preparing active drug and placebo formulations per manufacturing guidelines. Prior to administration via nasal spray, the dispenser was primed by depressing the nozzle until a full spray was emitted. Participants were asked to sit upright with their head tilted backwards before directing the spray nozzle to the back and center of one nostril. They were instructed to exhale and then inhale deeply while pressing the nozzle; after 10–15 s, they repeated the steps with the other nostril, alternating nostrils until they received the dose in its entirety.

### 2.4. Procedure

For ASD participants, study sessions proceeded as follows: (1) informed consent (first visit only); (2) baseline urine and plasma collection; 3) IN-OT/placebo dose administration; and (4) blood samples (~85 min) and urine (~120 min) collected post-administration. Collection time points were chosen based on reported peak level concentration windows [47,48]. In order to allow uOT to pool across the entire session period, we asked participants to refrain from urinating until the designated collection time. Participants who were unable to wait the full ~120 min interval were allowed to urinate and that collection time was noted; a second post-administration urine sample was then collected at the session end. AN and HC participants completed informed consent procedures at a screening visit prior to the first study session; during that visit, participants ate a standardized breakfast prior to IN-OT or placebo administration as a means to control for energy intake and hunger. Urine sampling protocols were identical to those implemented with the ASD group.

### 2.5. General Protocols: Urine and Plasma Oxytocin Levels

#### 2.5.1. Urine

Our urine collection protocol was designed to collect all urine produced (pooled) during the entire session to offset variability in pulsatile patterns of OT secretion. Urine was collected twice (i.e., once at baseline and once at the end of the session) per study session by having participants urinate into a receptacle (urine hat or cup). A disposable plastic pipette was then used to stir the sample and measure out two 2 mL and two 4 mL aliquots. Dry ice was used to snap-freeze each aliquot, and samples were then placed in a −80 °C freezer manufactured by Stirling (Athens, OH, USA) for long-term storage. When preparing to assay the samples, aliquots underwent controlled thawing and urine samples were subjected to solid-phase extraction (SPE) using SepPak C18 cartridges (cat no. WAT023590, Waters, Milford, MA, USA) to remove potential contaminants [38]. In order to account for variability in participants’ daily intake of fluids, we first measured levels of creatinine in each urine sample and then adjusted for the hormonal concentration ([OT]/[creatinine]), yielding uOT levels reported as the OT-to-creatinine ratio (pg/mg creatinine). Enzyme-linked immunosorbent assay (ELISA) procedures were implemented [49] using ELISA kits (Assay Designs, Inc./Enzo Life Sciences, Ann Arbor, MI, USA) as described previously [39,50,51,52].

#### 2.5.2. Plasma (ASD Participants Only)

Samples of 10 mL of collected blood were centrifuged using a 4 °C machine (3000 rpm) manufactured by Eppendorf (Newfield, CT, USA) for 15 min; plasma was aliquoted separately from the remaining blood products and placed into a −80 °C freezer for storage. When ready to assay, samples were thawed and purified by SPE. Plasma samples of 1 mL were eluted with 1 mL 80% acetonitrile, and 300 mL of ethanol was added to ensure proteins were denatured. Samples were then dried and reconstituted in an assay-appropriate buffer prior to pOT quantification [53]. Extracted samples were analyzed using ELISA kits (Assay Designs, Inc./Enzo Life Sciences, Ann Arbor, MI, USA), which have been validated in both plasma and urine media [37,39,40,51,52,54,55].

### 2.6. Data Analyses

Since the peripheral measures (uOT, pOT) collected in our project have both served as proxies for quantifying effective administration uptake of intranasal drugs, for these analyses, we aimed to determine whether assaying oxytocin levels from urine or plasma sampling would be more useful as downstream markers of IN-OT dosing [56]. The researcher (AT) who conducted all data analyses was blinded to which group received active drug versus placebo at each time-point in order to investigate whether group assignments could be ascertained by change in OT values alone, thus providing an indicator of the efficacy of both plasma and urine approaches. In order to maintain blinding, the active drug data points were labeled as “Randomization Group A”, while the placebo data points were labeled “Randomization Group B” by an unblinded researcher. Results from blinded analyses were subsequently reviewed by unblinded researchers to evaluate accuracy of our backward-chaining approach. Using SPSS 25.0 (Statistical Package for Social Sciences, Version 25, IBM, Armonk, NY, USA), Shapiro–Wilk and Kolmogorov–Smirnov tests were conducted to assess data normality. We found that uOT and pOT levels were not normally distributed; hence, uOT and pOT values were log-transformed before subsequent parametric statistical testing. Data normalization methods corresponded to those described in Hammock et al. (2012) [57]. Analyses of covariance (ANCOVAs) were applied to test the effects of Randomization Group (Drug, Placebo) on change in log(OT) (pre–post-drug administration), with age, sex, and visit order as covariates. For datasets that included AN, HC, and ASD participants, we also examined the effects of diagnostic groups.

### 2.7. Cluster Analyses

Two-step cluster (TSC) analyses were applied to determine whether Randomization Group assignment could be identified by change in OT alone for both uOT and pOT measurements. TSC analysis procedures employed a log-likelihood distance measure, Schwarz’s Bayesian (BIC) clustering criterion, and a maximum of 15 clusters. Silhouette coefficients of cohesion and separation, along with membership variables of each cluster solution, were used to assess whether outcome measurements served as accurate indicators of Randomization Group.

## 3. Results

Final blinded analyses of uOT data included N = 67 data points across all participants; final analyses of pOT data included N = 46 data points; we retained 44 data points with both uOT and pOT measurements. In total, three data points from the ASD group, three from HC, and five from AN were removed from final analyses due to assay issues (e.g., OT nondetectable, OT value above upper limit, OT value below lower limit, and creatine value above upper limit).

**Figure 2:**
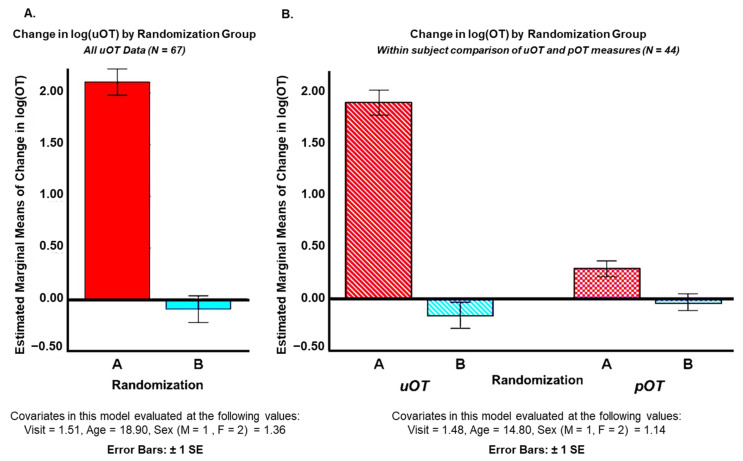
Mean differences between pre- and post-intranasal drug administration levels of (**A**) urine oxytocin (uOT) across all participants (ASD, AN, HC) and (**B**) mean differences between pre- and post-intranasal drug administration levels of uOT and plasma oxytocin (pOT) for ASD participants only. Analyses were conducted blind to which group (Randomization Group A or B) received active drug versus placebo at each time-point.

Covarying for visit order (1,2), age (years), and sex (M = 1, F = 2), analyses of log(OT) values showed significantly higher levels of post-OT in Randomization Group A than B for all uOT samples (N = 67) [F(1.62) = 147.314, *p* = 0.000, η^2^ = 0.71] and for corresponding uOT [F(1.39) = 137.109, *p* = 0.000, η^2^ = 0.78] and pOT [F(1.39) = 8.426, *p* = 0.006, η^2^ = 0.18] measures (N = 44). These findings clearly indicate that Randomization Group A received the active drug, while Randomization Group B received the placebo. However, it is notable that, while all comparisons reached statistical significance, the effect sizes for urine measures were considerably higher than the effect size for plasma measures (Figure 2).

**Figure 3:**
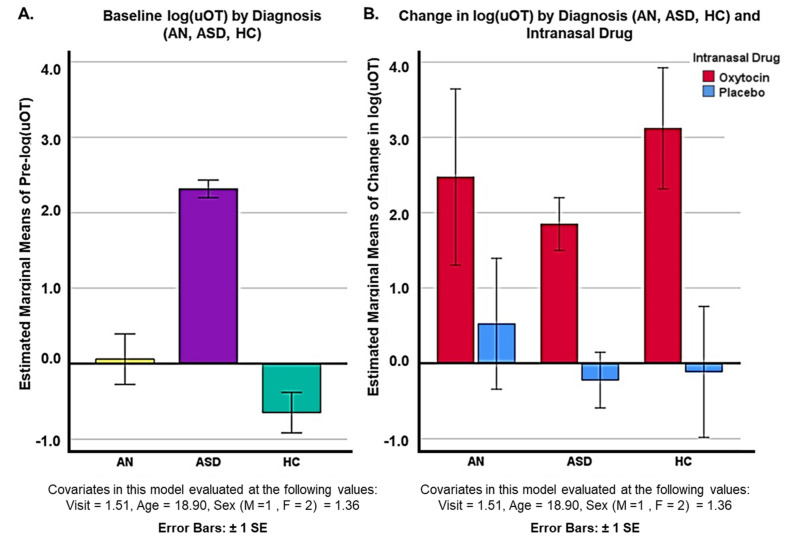
Unblinded comparison of (**A**) baseline uOT levels for diagnostic groups (AN, ASD, and HC) groups and (**B**) change in log(uOT) for diagnostic groups (AN, ASD, and HC).

Post hoc, unblinded uOT analysis of drug (oxytocin/placebo) and diagnostic (AN, ASD, and HC) groups revealed different baseline levels of uOT across groups. Pairwise comparisons adjusted for multiple comparisons (Bonferroni) showed that pre-log(uOT) levels for ASD participants (N = 49; mean = 2.475 ± 0.546) were significantly higher than pre-log(uOT) levels for AN participants (N = 9; mean = −0.536 ± 0.714; *p* = 0.000) and the HC group (N = 9; mean = −0.915 ± 0.635; *p* = 0.000) after covarying for visit, age, and sex. Baseline uOT levels for AN participants were also higher than HC participants (*p* = 0.0036) (Figure 3A). However, pairwise comparisons for change in uOT levels between each diagnostic group were not significantly different, indicating that all participants metabolized the drug regardless of initial levels (Figure 3B). However, interpretation of these data is limited by the discrepancy in sample sizes across groups.

**Figure 4:**
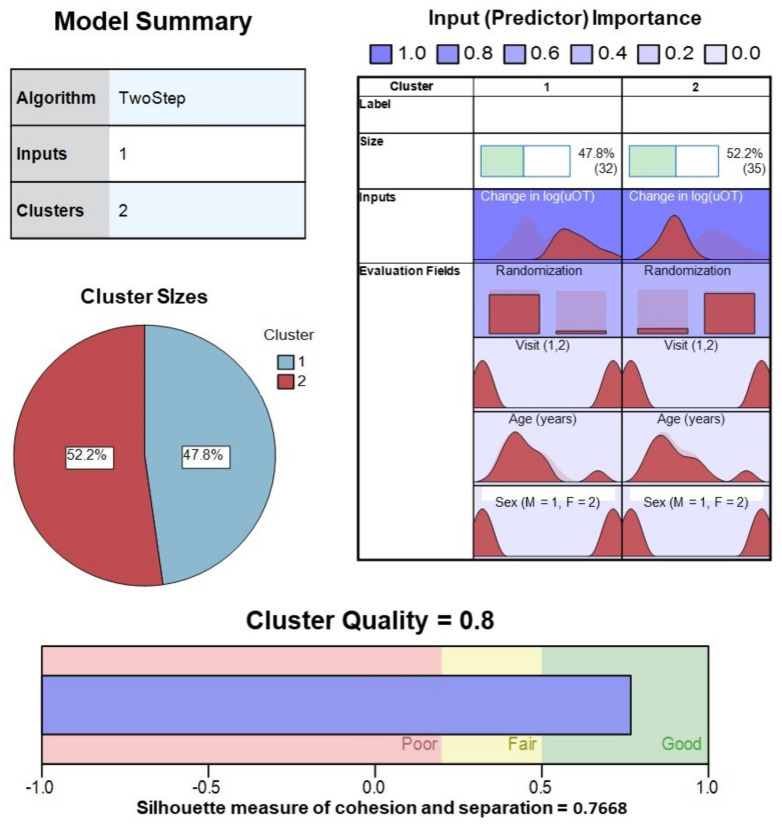
Model summary and cluster quality of two-step cluster analysis of pre–post-drug administration sampling of urine oxytocin from all participants (ASD, AN, and HC). Change in log transformed values of uOT, as well as Randomization Group, visit, age, sex, were evaluated for contribution (predictor importance) to the final solution. Good cluster cohesion and separation for the 2-cluster solution was driven largely by mean changes in log(uOT), further verifying the accuracy of our blinded uOT analysis.

TSC analysis yielded a two-cluster solution with a high silhouette measure of cohesion and separation coefficient (0.7668) for the change in log(uOT) data (N = 67). Cluster quality was in the good range (0.8) and differentiation of active drug versus placebo administration based on uOT values alone was strong (Figure 4). TSC analysis of change in log(pOT) data (N = 46) yielded a three-cluster solution with a silhouette measure of cohesion and separation coefficient of 0.6767 in the good range (0.7). For the sake of comparison, we also attempted to cluster log(pOT) data into two clusters (fixed), which resulted in a poorer quality (0.6) solution with a silhouette measure of cohesion and separation coefficient of 0.6471.

**Figure 5:**
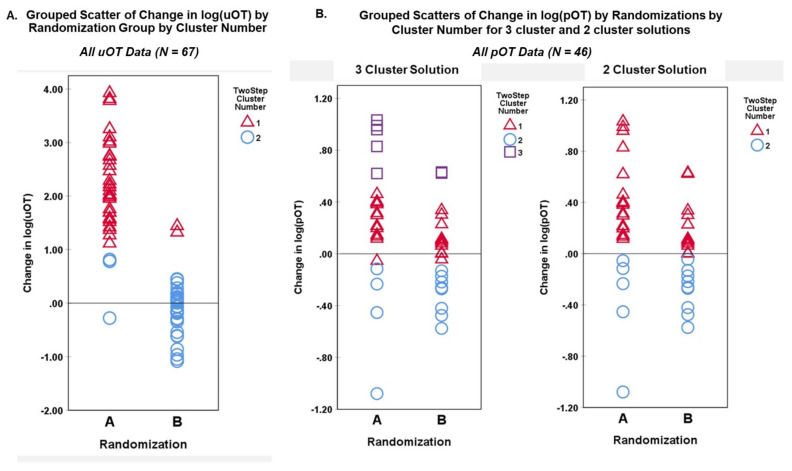
Grouped scatterplots of cluster membership variables for each TSC solution were plotted against the actual Randomization Group and log(OT) change for (**A**) urine and (**B**) plasma measures.

Grouped scatterplots of cluster membership variables for each TSC solution were plotted against the actual randomization group and log(OT) change for urine and plasma measures. Figure 5A illustrates the accurate correspondence of Randomization Group (A,B) to change in log(uOT) values (i.e., higher post-uOT in Randomization Group A and no significant pre–post-uOT change in Group B) supporting the efficacy and specificity of uOT as a methodological approach. Figure 5B shows that, while pOT assays can differentiate between pre–post-measures of pOT, the plasma approach captures lower IN-OT uptake (e.g., less overall OT detected) and is more susceptible to confounding variance.

## 4. Discussion

The primary objective of this paper was to evaluate the use of uOT sampling to assess endogenous oxytocin concentration following IN-OT administration to target behavioral changes in ASD and AN participants. Although the behavioral effects of OT are attributed to neural mechanisms, oxytocin concentrations and levels post-dosing are often measured through invasive means; blood draws for plasma are most common relative to the more invasive procedures used to assess CSF or direct cortical measures. Increasingly, studies have shifted to noninvasive techniques of collecting bio-samples (e.g., saliva and urine) for OT measures [58]. Because the precision of salivary OT measurement is confounded by exogenous OT remaining in the oropharynx following intranasal administration, we examined pre–post-drug change in endogenous OT levels from urine samples and compared results to a subset of participants who also had blood drawn near peak OT level times [59]. To date, few studies have directly compared the quality of uOT to pOT measurements as a physiological biomarker of response to IN-OT in intervention studies and treatment trials. Strengths of our study include our blinded and analytically novel approach to ascertain how well uOT compares to the more frequently measured pOT following intranasal administration, as well as our sample, which included two different clinical populations with noted rigidity and social concerns. Limitations of our study include a modest sample size and potential biological differences within the sample based on their disorders, although we attempted to account for these as covariates in our analyses (age, sex, and visit order) and addressed individual differences using a within-subjects repeated measures design. Moreover, although baseline levels of uOT differed across groups, mean change uOT values did not differ significantly by diagnostic group, thus confirming the effectiveness of IN-OT administration. It is worth noting that there may be differences in baseline OT levels for a range of factors, including biological and developmental age, sex/gender, time of day or sleep/rest/activity, medical and stress history, and other endogenous patterns of pulsative or triggered release. These potential differences, along with diagnostic history, should be explored in future analyses to better understand how IN-OT can be most effectively administered considering individual and subgroup differences. With the low side effect profile of IN-OT, these findings must be replicated in a larger control sample, as well as other clinical samples. Nevertheless, the methodology, results, and samples described in this report may be informative and applicable to ongoing and future IN-OT clinical trials.

Our novel blinded backward-chaining TSC approach was able to differentiate when a participant received an active intranasal drug versus placebo by measuring levels of endogenous OT in urine, regardless of clinical group or cognitive and behavioral targets. Within the subset of participants who also provided pre–post-administration blood samples, we were able to compare uOT directly to pOT samplings. Whereas cluster analysis of uOT change yielded a highly coherent and cohesive two-cluster solution, TSC analysis with pOT data resulted in a less clearly defined three-cluster solution. Reduced separation of cluster groups in the pOT data suggest more heterogeneity and ambiguity in assay of OT from plasma. These data highlight the advantages of urine assay for both participants (noninvasive procedure) and researchers (more precise analyses); urine measures may be a more efficacious and convenient tool for investigators who investigate physiological biomarkers to identify responders or nonresponders, as well as magnitude of intranasal treatment effects. In contrast, whilst one prior study showed pOT and uOT measures to be directly correlated [36], another reported a lack of correspondence between the two measures [37]. Data from the present study indicate increased variability in pOT at a single time point; this variance may account for some of the inconsistent results and correlations in the extant literature. Additional discrepancies may be attributed to differences in time course and steady state in urine versus blood; each have different volume distributions and clearance processes. Of note, behavioral outcomes in response to IN-OT and other neuropeptides often correlate with CSF changes measured 10–120 min after intranasal administration [60,61]. OT changes in blood and CSF plasma occur on the time course of minutes, whereas the changes reflected in urine capture outcomes over hours (Figure 1).

Methodological and ethical barriers limit our ability to directly access the brain and CSF for research; as such, efforts to elucidate the complex mechanisms subserving observable cognitive or behavioral outcomes in response to drug delivery have been impeded. Recently, a meta-analysis of 17 studies demonstrated a positive correlation between central and peripheral OT levels after IN-OT administration in human as well as animal studies [56]. In addition to the exogenous IN-OT findings, a positive correlation was also observed when the experimental design included measurements before and after a stressor task. Conversely, central and peripheral measures (csfOT versus pOT) did not correlate at baseline periods or single randomly selected time points in the same participants. Importantly, the mechanisms involved in how IN-OT targets behavioral changes through the brain remains unclear. The extant literature has long considered the blood–brain barrier impenetrable to OT, although alternative access to the brain has been shown to occur transmucosally through olfactory or trigeminal nerve pathways [4,27]. The higher correlation of peripheral and central OT levels after IN-OT administration may occur via increased hypothalamic release of OT into the periphery or exogenous OT reaching both CSF and peripheral circulation via nasal capillaries. Critically, however, one recent finding indicates that OT may, in fact, be transported from peripheral blood to the brain via the receptor for advanced glycation end-products (RAGE) in endothelial cells at the blood–brain barrier [62,63]. Further elucidation of the role of transport and carriers in the brain and body are essential for our understanding of IN-OT mechanisms of action. For additional consideration, see social behavior outcomes and peripheral level comparisons of OT released after intranasal IN-OT and parenteral MDMA when they are investigated in the same adult humans [47].

The potential application of a vasopressin, a neuropeptide hormone closely related to OT, is also of increasing interest for interventions in neurodevelopmental and neuropsychiatric disorders. Vasopressin is currently being investigated in autism, schizophrenia, pain perception, attention, and general social behavior [16]. In one published autism trial, chronic intranasal arginine vasopressin (AVP) was found to positively impact social responsiveness in children with ASD [64]. Intranasal AVP has also been cited for its analgesic effects [65,66,67] and its role in social attention and facial-recognition eye-tracking in healthy adults [68]. Desmopressin, a synthetic form of vasopressin, was initially designed as an oral drug for peripheral disorders to be used instead of brain-behavior treatments; intranasal Desmopressin is currently available as a treatment for hemophilia (clotting promoter; brand name: Stimate manufactured by Ferring GmbH (Kiel, Germany) for CSL Behring LLC (King of Prussia, PA, USA) and nocturnal polyuria (antidiuretic; brand name: Noctiva, manufactured by Renaissance Lakewood LLC (Lakewood, NJ, USA) for Serenity Pharmaceutics LLC (Milford, PA, USA).

Besides neuropeptides (e.g., OT and AVP), intranasal administration of neurohormones has also included sex hormones, such as estradial [69,70,71], progesterone [72,73,74], and testosterone [75], as well as growth hormones. Chronic intranasal administration of hexarelin, a synthetic growth hormone-releasing peptide, has been shown to promote growth in short children [76]. Intranasal administration of gonadotropin-releasing hormone agonists, including buserelin and nafarelin, have been shown to reduce symptom severity in women with endometriosis [77]. Though not a neurohormone, intranasal insulin has also been widely studied in CNS disorders because of its ability to pass through the blood–brain barrier [31,78,79] and the healthy brain’s significant requirements for glucose. Moreover, in trials of both healthy adults and patients with Alzheimer’s disease or cognitive impairment, intranasal administration of insulin versus placebo improved recall significantly [31,80].

## 5. Conclusions

Administration of drugs by the intranasal route is not without disadvantages. For example, successful delivery may be precluded if individuals have nasal congestion (e.g., from colds or allergies) and overuse may result in mucosal issues and increased risk for infection or anosmia. Further, some drugs may still degrade in the environment of the nasal cavity or have molecular weights prohibiting efficient absorption; dosage delivery amounts may also be more variable than oral, transdermal, or nebulized administrations. However, numerous advantages have also been identified for intranasal delivery of a wide variety of drugs and hormones (e.g., convenient, noninvasive, and rapid uptake), contributing to its increasing incorporation into clinical trials and practices. Drug delivery through the nose is afforded the benefit of highly permeable nasal mucosa and rich vasculature that facilitate drug absorption. Moreover, while neurohormones, such as OT, may not pass through the blood–brain barrier readily, they can still access CNS and trigger peripheral release without systemic side effects. Moreover, due to chemical half-life restrictions and temperature-related instability, some pharmaceuticals are not available in oral formulations. Unlike parenteral delivery systems, intranasal drugs do not have to enter systemic circulation or undergo hepatic-gastrointestinal “first pass” elimination. In order to prescribe optimal doses for targeted therapeutic effects, blood levels are important to obtain for some parenteral drugs and these procedures increase patient burden. However, our data show that, when employing intranasal drugs, urine concentration levels collected through noninvasive means may serve as a favorable biomarker to ascertain successful and sufficient administration with less burden. In addition, urine level measurements may be pragmatic because intranasal delivery often results in rapid peaks and clearance with nonmodified synthetic neuropeptide hormones.

Our work provides strong support for the utility of intranasal drug delivery of neurohormones; future work will need to continue investigating mechanisms of action and efficacy, as well as determining whether urinary output levels (e.g., area under the curve) do correlate with targeted treatment outcomes studied in intranasal drug trials. Furthermore, given the rapid-acting attributes of intranasal delivery, long-acting formulations will need to be developed for neurohormones targeting protracted time frames (e.g., full work or school days) unless administration protocols are designed for shorter intervals. In light of the increasing prevalence rates of ASD, AN, and other NDDs and neuropsychiatric disorders, development of efficacious treatments that are accessible widely, particularly for vulnerable populations, should be a priority.

## Data Availability

Not applicable.
